# Characteristics of the Arcing Plasma Formation Effect in Spark-Assisted Chemical Engraving of Glass, Based on Machine Vision

**DOI:** 10.3390/ma11040470

**Published:** 2018-03-22

**Authors:** Chao-Ching Ho, Dung-Sheng Wu

**Affiliations:** 1 Graduate Institute of Manufacturing Technology and Department of Mechanical Engineering, National Taipei University of Technology, 1, Sec. 3, Zhongxiao E. Rd., Taipei 10608, Taiwan; 2 Department of Mechanical Engineering, National Yunlin University of Science and Technology, Yunlin 64002, Taiwan; m10211002@yuntech.org.tw

**Keywords:** in situ estimation, SACE-drilled hole depth, spark-assisted chemical engraving, glass machining, computer vision, electrochemical discharge machining

## Abstract

Spark-assisted chemical engraving (SACE) is a non-traditional machining technology that is used to machine electrically non-conducting materials including glass, ceramics, and quartz. The processing accuracy, machining efficiency, and reproducibility are the key factors in the SACE process. In the present study, a machine vision method is applied to monitor and estimate the status of a SACE-drilled hole in quartz glass. During the machining of quartz glass, the spring-fed tool electrode was pre-pressured on the quartz glass surface to feed the electrode that was in contact with the machining surface of the quartz glass. In situ image acquisition and analysis of the SACE drilling processes were used to analyze the captured image of the state of the spark discharge at the tip and sidewall of the electrode. The results indicated an association between the accumulative size of the SACE-induced spark area and deepness of the hole. The results indicated that the evaluated depths of the SACE-machined holes were a proportional function of the accumulative spark size with a high degree of correlation. The study proposes an innovative computer vision-based method to estimate the deepness and status of SACE-drilled holes in real time.

## 1. Introduction

Non-traditional engraving processes have been becoming a promising technology for micro-machining. New groups of unconventional engraving processes such as the sustainable biomachining of metals by using bacteria [1,2,3] and spark-assisted chemical engraving of non-conductive materials [4] can remove material from a workpiece without affecting the mechanical properties of the material due to the lack of machining forces produced in the process. Spark-assisted chemical engraving (SACE) is also known as electrochemical discharge machining (ECDM) and is a non-traditional machining process that is advantageous for machining non-conducting [5], hard and brittle materials with low residual stress to produce micron-sized holes. In the field of electrochemical discharge machining, it is important to focus on enhancing the processing efficiency, hole quality, and reproducibility. The processing efficiency is improved if the state of the SACE machining is monitored immediately, and this improves the quality of the processing holes and reduces the processing cost. However, the machining of non-conducting materials with the SACE machining process is associated with challenges [6]. A literature survey indicates that several attempts have focused on improving the machining performance of SACE [4,5,6].

The gas film thickness is the main limiting factor of electrochemical discharge phenomena. Surfactants were added to an electrolyte to increase the wettability of the tool electrode and to thereby reduce the gas film thickness. It was experimentally observed that the critical voltage was significantly reduced due to the increase in the wettability of the tool electrode, and the gas film thickness was thereby reduced by adding surfactants to the electrolyte. In 2007, Zheng et al. [7] changed the electrode geometry to reduce the impact area of the hole discharge and improve the wettability of the electrode to reduce the thickness of the gas film. The combination of the flat sidewall and flat front tool produced a compact gas film attached to the tool electrode. Additionally, the experiment confirmed that this improved the processing accuracy and reduced the entrance reaming problems. In 2010, Cheng et al. [8] suggested that the stability of the gas film formation determined the machining accuracy, surface roughness, and reproducibility of the machined parts and indicated that a correlation existed between the current signal and the gas film quality. An increase in the processing deepness beyond 300 μm made it difficult to maintain the machining efficiency due to the worsening of the electrolyte circulation at the tooltip and the increase of the average current value. In 2011, Jiang et al. [9] established a finite element model to correlate spark energy and the geometry of removed material. However, uncertainties were involved in the discharging activity during ECDM due to the high instability of the gas film. In 2015, Gouda et al. [10] reviewed the parameters that influence the material removal rate for electrochemical discharge machining and concluded that parameters including DC power supply, tool electrode, electrolyte, and work-piece materials affect the machining performance. The parameters also contributed to the stability of the gas film. During the ECDM process, an unstable gas film around the tool electrode reduces machining reproducibility. In 2016, Giandomenico et al. [11] indicated that the disadvantage of the electro-discharge machining process includes relatively poor accuracy due to gas bubbles that increase the current density at the side gap.

Investigations have been conducted to identify the main parameters that influence SACE machining. Thus, there is a paucity of studies that offer a complete understanding of the machining process. During the SACE machining, spark discharges induced inside a gas film surround the tool electrode. Spark discharges occur between the tool electrode and the electrolyte when the voltage between the electrodes exceeds the critical voltage. Previous studies [12,13] indicate that the gas film is a key factor for spark discharge and that the quality of machining performed by spark discharging is essentially controlled by the quality of the gas film during the SACE process. The quality of the gas film is a dominant factor that determines the machining qualities. Although the machining mechanism of SACE is not established to date [14], it is generally agreed that the main machining process is the thermal melting of the workpiece. However, active feedback monitoring of the process is needed to further improve the machining characteristics of SACE.

**Figure 1:**
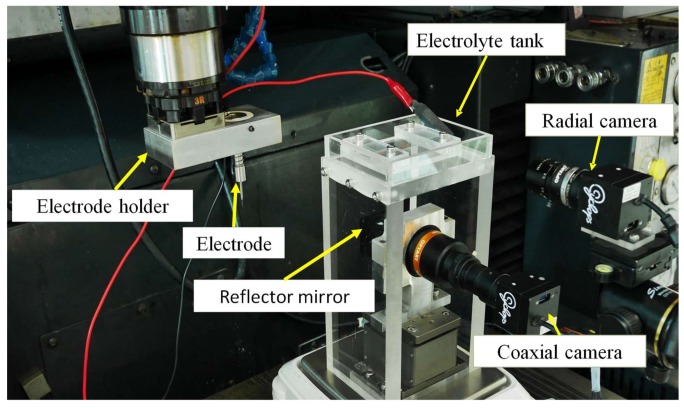
The experimental arrangement for spark assisted chemical engraving drilling.

In this study, we propose an innovative monitoring method for the in-situ measuring of SACE-drilled holes based on computer vision analysis. As a result, a low cost in-situ optical inspection system was developed to characterize the spark discharge during SACE machining. The monitoring of spark discharge is used as the basis for an in situ measuring system for SACE hole drilling. This monitoring arrangement includes image acquisition and pre-processing units, a SACE drilling apparatus, and a computer analysis unit as presented in Figure 1. First, we employed the industrial camera of the image acquisition and pre-processing unit for the in situ acquisition of an image of the SACE drilling region of the surface of the workpiece. The high spark energy caused the melting and evaporation of the material during the SACE drilling and resulted in thermal erosion due to the heat simultaneously generated by the discharges. The sparking action phenomenon involved arcing action, and this is captured by the industrial cameras. The images comprised of light emission observed in the film in which electrical discharges occur between the tool electrode and the surrounding electrolyte. Finally, image data are analyzed at the computer analysis unit to conduct an in situ processing of the relationship between the geometrical forms of the drilling region comprising of the deepness of a blind hole and image information. The experimental arrangement realizes benefits including lower costs, in situ measurement, and improved performance.

## 2. Experimental Setup and Method

### 2.1. Setup

**Figure 2:**
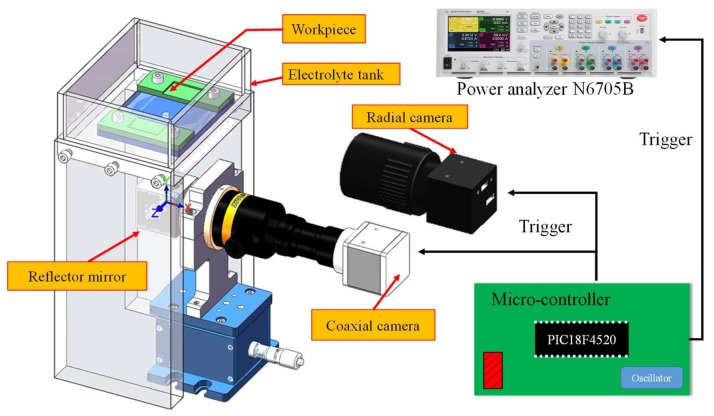
The schematic diagram of the spark-assisted chemical engraving (SACE) experimental setup.

Figure 1 shows the experimental arrangement comprising of the SACE drilling module, the power supply, the image acquisition and pre-processing unit, and the computer control and analysis system. A schematic diagram of the SACE experimental setup is illustrated in Figure 2. The computer is linked to a programmable intelligent computer (PIC) microcontroller (Microchip Technology Inc., Chandler, AZ, USA) that controls the SACE drilling parameters including the drilling voltage and current to trigger and control the SACE drilling module. The workpiece was dipped in a bath that contained a solution with a 5 M KOH (the electrolyte solution), and a tool electrode was positioned above the workpiece surface (quartz glass, 1.0 mm thick). The electrolyte bath served as the machining chamber, the attachment to supply power to the electrode tool, a spring-fed tool arrangement, and a DC power supply (the programmable DC power analyzer Agilent N6705, Santa Clara, CA, USA). During the machining of quartz glass, the spring-fed tool electrode was pre-pressured on the quartz glass surface to feed the electrode that was in contact with the machining surface of the quartz glass. The spring-fed tool arrangement was designed and fabricated to enable the smooth feeding of the electrode tool and to avoid mechanical breaking action that would break the quartz glass.

**Figure 3:**
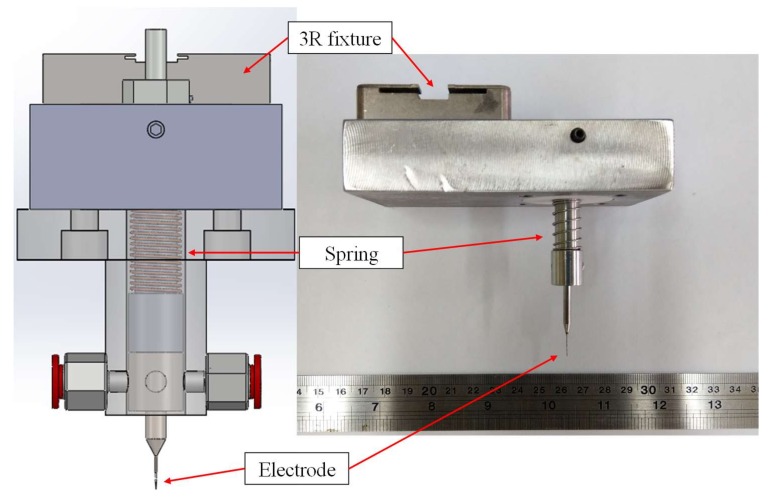
The tapered tool electrodes with a spring-fed mechanism.

**Figure 4:**
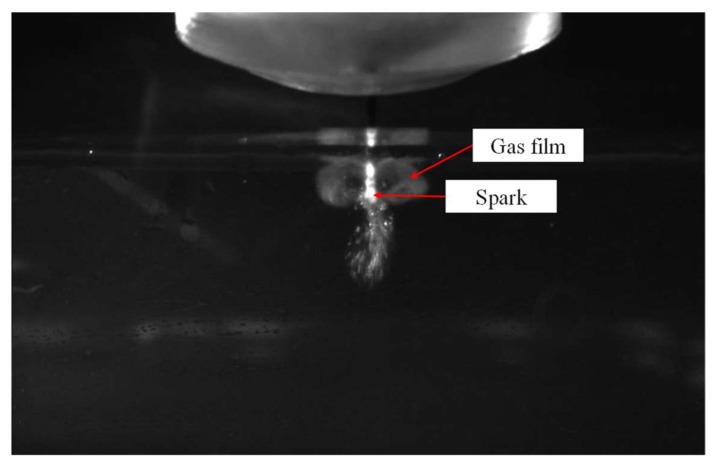
The appearance of the sparking phenomenon is observed when the gas film is formed.

The application of a suitable electric potential (i.e., 40 V in our system) across the two electrodes led to the construction of the gas film around the tool electrode, and the arcing plasma induced by the discharge phenomenon that directly followed contributed to the material removal. Figure 3 shows the image of the gas film formation by using a tapered tungsten carbide (WC) electrode with a diameter of 249 μm that is arranged based on the tool immersion depth. The tapered tool electrode design (i.e., complex shaped electrodes [15]) is able to help to drill deep and narrow cavities [16] and stabilize the machining process [17]. A tapered tool electrode was clamped on the chuck and employed in the experiments to increase the consistency of spark generation and to focus the discharges on a concentrated region. The tool and chuck were connected to the power supply during the SACE process. When the DC voltage was applied, a spark discharge occurred primarily at the electrolyte surface as well as at the tooltip and the electrolyte surface exhibited a concave shape due to the surface tension. As shown in Figure 4, the presence of a bubble layer was observed around the electrodes and it coalesced into a gas film, thereby leading to the expected light emission (i.e., the sparking phenomenon).

The arcing plasma image was obtained using the two complementary metal–oxide–semiconductor (CMOS) cameras. One of the cameras was mounted radially 190 mm from the work site with a 35 mm focus lens and a 1.5× close-up lens. The other camera was mounted coaxially at a distance of 100 mm below the electrolyte tank with a 0.7× telocentric lens. The PIC microcontroller unit triggered the SACE power module to produce a single SACE voltage pulse and instantaneously delayed it for a short time period in order to trigger the image acquisition and pre-processing unit. This captured each single arcing plasma image of the sparking phenomenon. The surroundings settings and drilling parameters of the experiment are listed in Table 1.

### 2.2. Estimation Method

**Figure 5:**
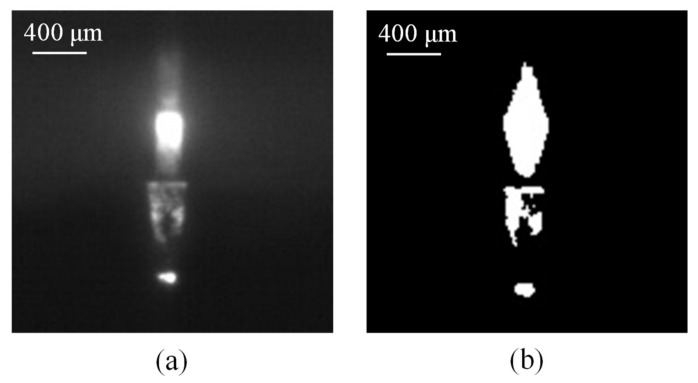
The arcing plasma area of an image of quartz glass in the radial direction (**a**) acquired by an industrial camera and (**b**) after the thresholding technique.

**Figure 6:**
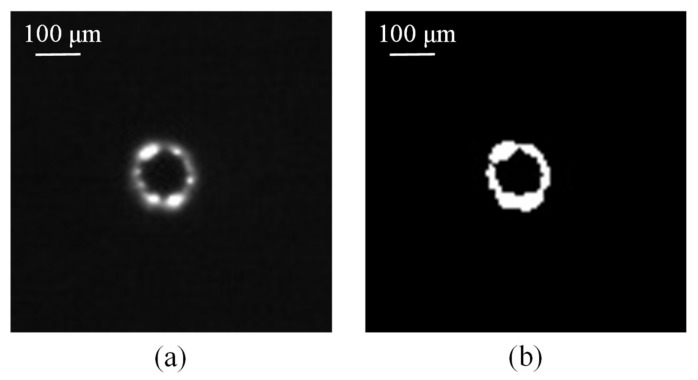
The arcing plasma area of an image of quartz glass in the coaxial direction (**a**) acquired by an industrial camera and (**b**) after the thresholding technique.

The SACE-drilled system comprising of the blind hole deepness analysis were acquired and evaluated using 8-bit encoding and 752 × 480 pixel images. We used the thresholding technique during the image processing to calculate the arcing plasma emission area and compute the entire pixel area of the arcing plasma region. The images of the quartz glass after the thresholding technique are shown in Figure 5 in the radial direction and Figure 6 in the coaxial direction. The machining time is used to estimate the deepness of a SACE-drilled hole in the conventional control method. An increase in the machining time increased the deepness of the hole. The conventional control method used experience-based control strategies to sense the finalization of the drilling process off-line.

The computer vision method was used to determine the correlation between the SACE-induced arcing plasma and the SACE-drilled hole deepness. It is an innovative evaluation mechanism to determine the deepness of a SACE-drilled hole during drilling. During the SACE-drilled machining, a spark discharge is usually induced as a result of the melting and evaporation of the material from the arcing-heated region. The electrical spark discharges occurred through a gas film constructed around the tool electrode and were observed by the setup of the camera in the radial and coaxial directions. The pixels of the SACE-induced plasma area for each individual single image frame from the start to the end of the drilling were calculated using machine vision processing. We subsequently accumulated the entire pixel area for single image frames of the plasma area through SACE drilling and obtained the whole pixel area of the plasma area from the start to end.

## 3. Results and Discussion

**Figure 7:**
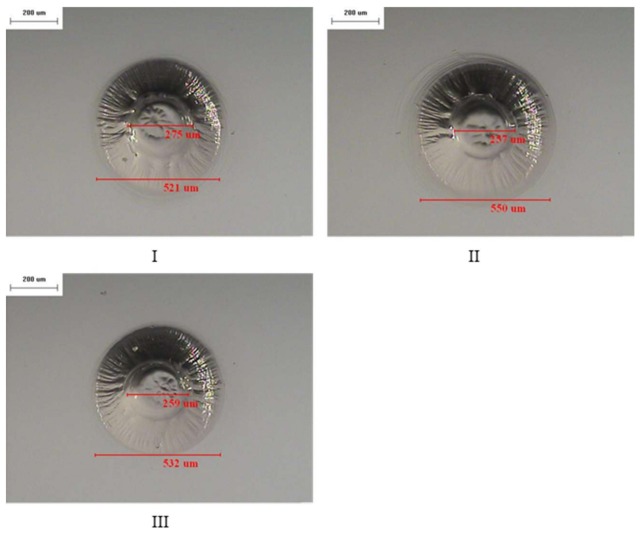
The diameter of the holes and the area of the thermally affected region are investigated by optical microscopy (OM) (**I**–**III**) 50×.

In these experiments, the drilling deepness was accounted for using a load-cell based mechanical scaler to establish the processing depth. The diameter of the holes and the area of the thermal-affected region were investigated using optical microscopy (OM) as shown in Table 2 and Figure 7. The results indicated deviations in the diameter of the machined contour and the thermal-affected region of approximately 3.7% and 2.7%, respectively. The experimental results indicated good agreement with those obtained by extant studies [18]. In reference [18], Wüthrich et al. indicated that deviations of the micro-holes with a mean diameter of a few hundred microns typically corresponded to approximately 20% of perfect circular contour.

**Figure 8:**
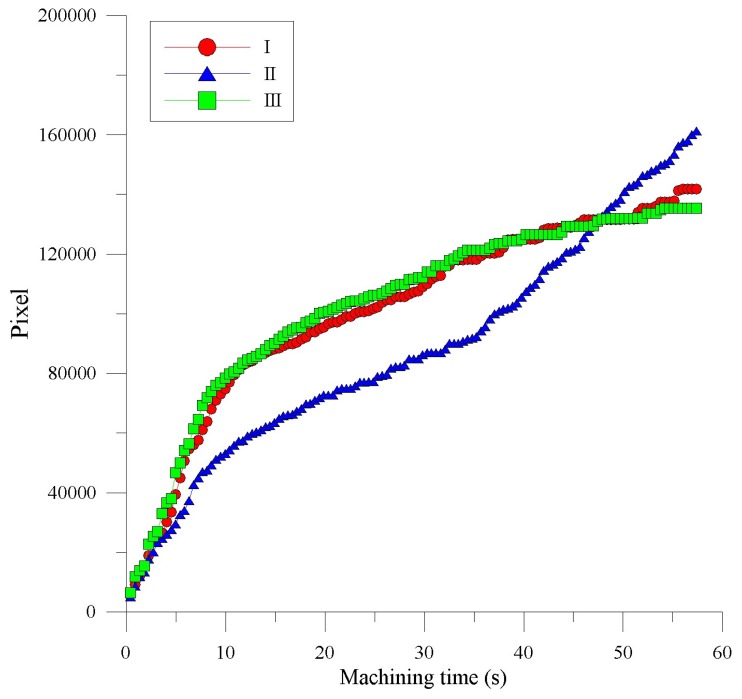
The accumulated light emission pixels of the SACE-drilled holes, acquired by using a coaxially mounted industrial camera.

**Figure 9:**
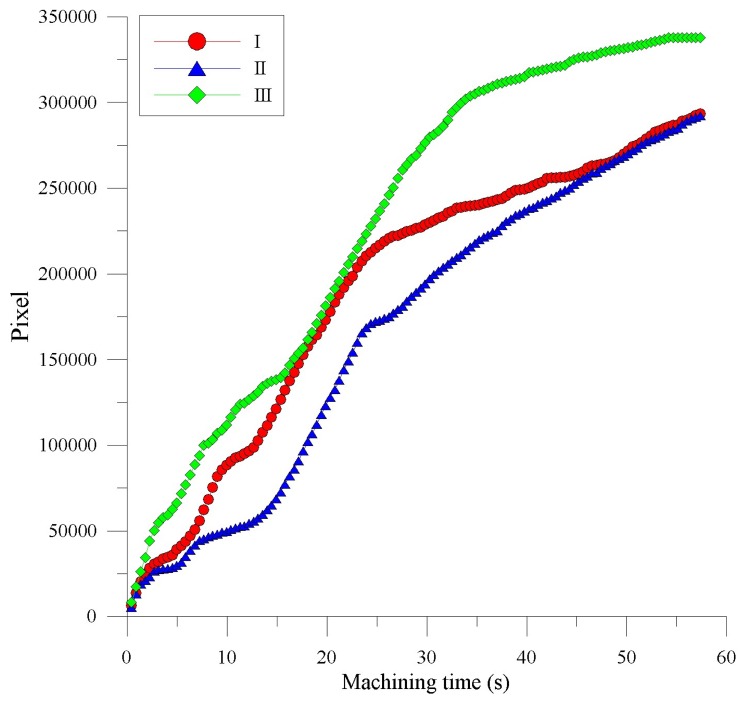
The accumulated light emission pixels of the SACE-drilled holes, acquired by using a radially mounted industrial camera.

The resulting light emission images in the radial and coaxial directions for SACE-drilled machining were obtained using a process time of 60 s in which the machining energy of the SACE was 40 V and 1.5 A for quartz glass as shown in Figure 8 and Figure 9, respectively. The results suggested that a certain degree of deviation continued to exist in the light emission pixels. This was caused by the bubbles produced at the front end of the electrode and also around the electrode sidewall [19]. Therefore, a significant spark light emission was observed around the electrode. The unstable gas film fluctuated arbitrarily and randomly, and this resulted in irregular spark behavior [20].

**Figure 10:**
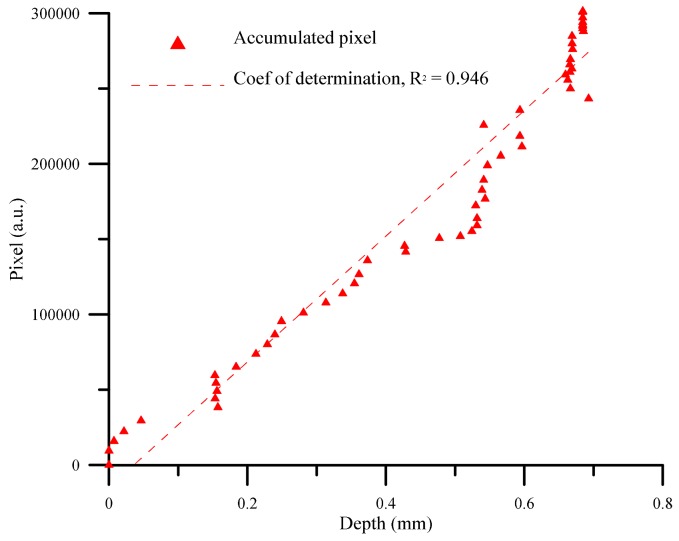
The correlation between the deepness of the SACE-drilled hole and the pixels of the arcing plasma area, acquired by using a radially mounted industrial camera.

The accumulated light emission pixels of the SACE-drilled holes evidently increased with increases in the drilling time. The correlation between the drilling-hole deepness and the pixel value of the SACE-induced plasma area is presented in the radial direction in Figure 10. The results indicate that the deepness of the SACE-drilled holes was estimated by a monotonically increasing function.

**Figure 11:**
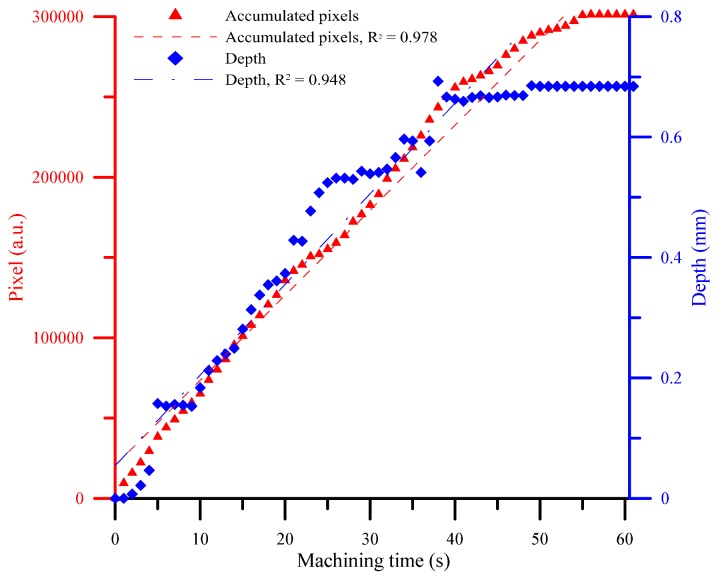
The correlation between pixels and deepness relative to the drilling time.

The deepness of the SACE-drilled hole was investigated as a function of the accumulative plasma size to fully monitor the process parameters. Figure 10 shows the deepness as a function of the machining time at which the camera detected the arcing plasma pixel. The results revealed that the accumulative arcing plasma size exhibited a high coefficient of determination (R^2^) of 0.946 with respect to the machining depth. The experimental results indicated a good agreement [21] with those obtained by SACE machining, and this was caused mainly by spark discharges that occurred through a gas film constructed around the tool electrode. As shown in Figure 11, the hole deepness is proportionally related to the drilling time and the total number of the pixels of all the frames of the plasma area. Based on the proportional relation, we used the entire accumulative pixel values to evaluate and sense the SACE-drilled hole deepness. In this study, we calculated that the size of the SACE-induced plasma area at the surface of the workpiece can be converted into the value of the pixels. The spark energy conditions determined both the SACE-drilled hole deepness and size of the plasma area relative to the drilling time. Thus, we analyzed and obtained the entire pixel area of the plasma area in situ during processing. Additionally, we realized the in situ monitoring and evaluation of the deepness of a SACE-drilled hole. Figure 11 shows the relationship between pixels and deepness relative to the process time. The linear dependency of the two variables revealed the association between the measured spark pixel and drilled depth. In this case, it is observed that the machined deepness was saturated at a time corresponding to 50 s at an approximate deepness of 0.6 mm. The accumulated spark pixel also stopped increasing at the end of the machining stage. It was observed that the R^2^ of the linear regression between the accumulative arcing plasma size and the machining time is 0.978. Furthermore, the R^2^ of the linear regression between the machined deepness and the machining time is 0.948 before the saturation depth. The results also indicated that the machined status was estimated using the acquired image pixel.

## 4. Conclusions

This study examined a novel in situ technique to estimate the depths of SACE-drilled holes. In this study, visual signals that could be used for the on-line monitoring of SACE machining were discussed first. The vision signal was directly accessible for the measurement and was therefore specifically discussed. Based on the SACE-induced arcing plasma area that is converted into a pixel value, we obtained an association between the accumulative pixels and hole depth. The system acquired and analyzed images of SACE drilling in real time and, thus, the hole deepness evaluation was also in real time. The evaluation mechanism based on a machine vision processing method was combined with a deepness control method to perform conventional SACE machining and to realize increasingly accurate deepness control for a SACE-drilled hole. The key deepness information on the machining process was accessible through a relatively simple analysis without requiring additional sensors. As ECDM is a spark-based and highly stochastic micromachining method, future work will focus on further investigation into correlating the spark pixel with the effects of various process parameters such as applied voltage, electrolyte concentration and tool geometry on machining status.

## Figures and Tables

**Table 1:** The experimental parameters.

Parameter	Value
Voltage	40 V
Peak current	1.5 A
Workpiece material	Quartz glass
Thickness of workpiece	10 mm
Tool electrode material	Tungsten carbide
Electrolyte	KOH

**Table 2:** Hole diameter and diameter of the heat affected zone.

Experimental Dataset	Hole Diameter (μm)	Heat Affected Diameter (μm)
I	275	521
II	257	550
III	259	532
